# The Association between Portion Sizes from High-Energy-Dense Foods and Body Composition in European Adolescents: The HELENA Study †

**DOI:** 10.3390/nu13030954

**Published:** 2021-03-16

**Authors:** Sondos M. Flieh, María L. Miguel-Berges, Esther M. González-Gil, Frédéric Gottrand, Laura Censi, Kurt Widhalm, Yannis Manios, Anthony Kafatos, Dénes Molnár, Jean Dallongeville, Peter Stehle, Marcela Gonzalez-Gross, Ascensión Marcos, Stefaan De Henauw, Cristina Molina-Hidalgo, Inge Huybrechts, Luis A. Moreno

**Affiliations:** 1 Growth, Exercise, Nutrition and Development (GENUD) Research Group, Faculty of Health Sciences, University of Zaragoza, 50009 Zaragoza, Spain; sondosnserat991@gmail.com (S.M.F.); mlmiguel@unizar.es (M.L.M.-B.); lmoreno@unizar.es (L.A.M.); 2 Instituto Agroalimentario de Aragón (IA2), 50013 Zaragoza, Spain; 3 Instituto de Investigación Sanitaria Aragón (IIS Aragón), 50009 Zaragoza, Spain; 4 Center of Biomedical Research (CIBM), Department of Biochemistry and Molecular Biology II, Instituto de Nutrición y Tecnología de los Alimentos, University of Granada, 18071 Granada, Spain; 5 CHU Lille, University Lille, INSERM U1286 Infinite, F-59000 Lille, France; Frederic.GOTTRAND@chru-lille.fr; 6 Council for Agricultural Research and Economics, Research Centre for Food and Nutrition, 00178 Roma, Italy; laura.censi@crea.gov.it; 7 Department of Gastroenterology and Hepatology, Medical University of Vienna, 1090 Vienna, Austria; kurt.widhalm@meduniwien.ac.at; 8 Austrian Academic Institute for Clinical Nutrition, A-3100 Vienna, Austria; 9 Department of Nutrition and Dietetics, School of Health Science and Education, Harokopio University, 17671 Athens, Greece; manios@hua.gr; 10 Institute of Agri-Food and Life Sciences, Hellenic Mediterranean University Research Centre, 71410 Heraklion, Greece; 11 Faculty of Medicine, University of Crete, GR-71003 Crete, Greece; kafatos@med.uoc.gr; 12 Department of Pediatrics, Medical School, University of Pécs, H-7624 Pécs, Hungary; molnar.denes@pte.hu; 13 Department of Epidemiology and Public Health, Institut Pasteur de Lille, 59000 Lille, France; Jean.Dallongeville@pasteur-lille.fr; 14 Department of Nutrition and Food Sciences, University of Bonn, D-53115 Bonn, Germany; pstehle@uni-bonn.de; 15 CIBER Fisiopatología de la Obesidad y Nutrición (CIBEROBN), Instituto de Salud Carlos III, 28029 Madrid, Spain; marcela.gonzalez.gross@upm.es; 16 ImFINE Research Group, Department of Health and Human Performance, Faculty of Physical Activity and Sport-INEF, Universidad Politécnica de Madrid, 28040 Madrid, Spain; 17 Inmunonutrition Research Group, Department of Metabolism and Nutrition, Institute of Food Science, Technology and Nutrition (ICTAN), Spanish National Research Council (CSIC), 28040 Madrid, Spain; amarcos@ictan.csic.es; 18 Department of Public Health and Primary Care, Faculty of Medicine and Health Sciences, Ghent University, 9000 Ghent, Belgium; Stefaan.DeHenauw@UGent.be; 19 EFFECTS 262 Department of Medical Physiology, School of Medicine, University of Granada,18071 Granada, Spain; criismh@correo.ugr.es; 20 International Agency for Research on Cancer (IARC), 69372 Lyon, France; huybrechtsi@iarc.fr; 21 Department of Public Health, Ghent University, 9000 Ghent, Belgium

**Keywords:** energy dense food, food portion size, body mass index, fat mass index, obesity, adolescent, Europe

## Abstract

Obesity prevalence has been simultaneously increasing with high consumption of large food portion sizes (PS). However, there is scarce information on PS of energy-dense (ED) foods as a potential risk factor of obesity in adolescents. In the present study, we investigate the association between the PS of the most ED foods and body composition. A sample of 1889 adolescents (54.4% females) from the Healthy Lifestyle in Europe by Nutrition in Adolescence cross-sectional multicenter study (HELENA–CSS) study were included. Most ED foods (e.g., cheese) were selected according to higher fat and/or sugar content and low fiber and water. Linear and ordinal logistic regression models were adjusted for age, physical activity, total energy intake (TEI), and socioeconomic status (SES). Analysis was performed both in those adolescents reporting plausible energy intake according to the approach of Goldberg et al. and in the whole sample. In male plausible reporters, PS from “breakfast cereals” showed a significant and positive association with BMI (β = 0.012; 0.048). PS from “carbonated soft drinks” in males (OR = 1.001; 95% CI 1.000; 1.002) and “bread and rolls” in females (OR = 1.002; 95% CI 1.000; 1.004) were associated with higher probability of having obesity, while “sweet bakery products” were associated with lower probability of having obesity (OR = 0.996; 95% CI 0.991; 0.999) in females. The present study suggests association between PS of ED foods and obesity in European adolescents. Prospective studies are needed to examine the effect of prolonged exposure to large PS and obesity development.

## 1. Introduction

According to WHO reports, overweight and obesity prevalence in children and adolescents aged between 5 and 19 years continuously rose from just 4% in 1975 to over 18% in 2016, corresponding with over 340 million affected persons worldwide [1]. Youth obesity is classified as chronic, noncommunicable disease that could lead to acute as well as long-lasting health problems at a younger age [2], cardiovascular diseases [3], insulin resistance [4] and type 2 diabetes [5] and, since obesity tracks from youth to adulthood, a greater risk of early morbidity and premature mortality [6,7]. Adolescence is a critical period in which dietary habits are in transition into adulthood. Several studies suggested that adolescents with obesity tend to eat more ED foods compared with normal-weight adolescents [8].

Overall, weight gain is the result from an imbalance between daily nutritive energy intake and energy expenditure as a sum of resting energy expenditure and physical exercise [9]. Apart from increasing sports activities, there are several components of the food environment supporting energy overnutrition [9], food portion sizes (PS) being probably one of the most relevant factors [10]. A portion is described as the amount of food that we choose to eat for a meal or snack or the amount of a food that we decide to eat or serve to an individual at a single eating occasion [11]. Previous studies found that the PS of some prepacked foods, as well as menu sizes consumed in restaurants, have increased dramatically over the last 30 years, concurred with the recent increase in obesity prevalence [12,13]. Specifically, increased PS of foods commonly served in restaurants and market is considered as a major component of the food environment that contributes to the excess of energy consumption and the development of obesity in all age groups [10,14]. Even though PS have been increasing over time, its association with weight would be predictable. In children and adolescents, several short-term controlled feeding trials found that serving PS and the amount of energy consumed per meal were associated significantly with a higher body mass index (BMI) percentile in school-aged males and adolescents of both genders but not in the youngest children (3 to 5 years) [15,16,17].

Energy density (ED) refers to the energy amount in each weight of food and/or beverage (kcal/g) [18] and mainly depends on the fat and water content of the food [19]. The World Cancer Research Fund has classified food containing 60–150 kcal/100 g as low-ED foods, characterized by high water and fiber content. Medium-ED foods contain 100–225 kcal/100 g, and high-ED foods contain more than 225–275 kcal/100 g [20]. This classification is one of the most commonly used by several studies [11,21] to group specific food items by its energy content.

Previous epidemiologic studies in European adults found only a limited relationship between PS from ED foods and with the actual BMI [22]. An intervention study focusing on the effect of large PS on body weight by a midday meal manipulation in adults noticed that the weight changes were not significant over time or between test periods [23]. On other hand, studies in young children found that PS and ED increased the energy intake at meals [24,25]. However, a cross-sectional study found that PS of milk, bread, cereal, juice, and peanut butter were associated with higher contribution to daily energy intake in children; moreover, they found that the PS z-scores were positively linked with both energy intake and body weight [26]. In the same vein, another study in children found that, when large portion of snack foods were consumed in the absence of hunger in females aged 5 and 7 years old, they had 4.6 times more probability of being overweight at both ages [27]. Finally, in British adolescents, PS of high ED foods from cream, high-fiber breakfast cereals, and soda were positively associated with a higher BMI [11].

Given the scarce previous literature, more information on the relationship between consumption of large food PS, specially of high-ED foods, and body composition are needed. Therefore, the aim of this study is to investigate the association between PS from most frequently consumed high-ED foods and obesity in a sample of European adolescents.

## 2. Materials and Methods

### 2.1. Study Design

The Healthy Lifestyle in Europe by Nutrition in Adolescence (HELENA) cross-sectional multicenter study (CSS) took place in 2006–2007 and aimed to evaluate the nutritional status of adolescent in Europe. The age of adolescents was between 12.5 and 17.5 years and were recruited from ten European cities: Heraklion and Athens (Greece), Ghent (Belgium), Dortmund (Germany), Rome (Italy), Pécs (Hungary), Stockholm (Sweden), Lille (France), Vienna (Austria), and Zaragoza (Spain) [28]. The HELENA–CSS basic objective was to obtain comparable and reliable data from selected European adolescent groups (*n* = 3528, 52.3% girls) using widely relevant health and nutrition-related parameters, including mensuration for anthropometric, physical activity, dietary information, choice and preferences of food, metabolism of lipid and glucose, vitamin and mineral serum status, physical fitness, and genetic indicator [29]. The inclusion norms for the main study were participants who were not involve concurrently in other clinical experiments, aged greater than 12.5 years and not exceeding 17.5, and finally, have not suffered from any acute illness less than 1 week before the inclusion procedure [30]. More detailed information about sampling and recruitment procedure were described elsewhere [30].

The Research Ethics Committees in each participant city approved this study, followed by the ethical guidelines of the Declaration of Helsinki 1964: revision of Edinburgh 2000 [31]; moreover, a written consent form was obtained from all participating adolescents and their parents [31].

### 2.2. Study Sample

From the total sample of the HELENA–CSS, about 1889 adolescents (54.4% females) were included in our study. The inclusion benchmarks were participants who have full two measurements of the 24-h recall and complete data for weight and height and represented (53.5%) of from the whole sample. In total, 140 adolescents were excluded, as they were classified as underweight, because there were too few in this category to provide adequate power. In this study, nutritional intake data from 8 European countries were included; however, data from Pecs (Hungary) and Heraklion (Greece) were not included, because only one 24-hr recall was available. The approach of Goldberg et al. [32] was used to classify adolescents to under-reporters (ratio of energy intake to basal metabolic rate <0.96) and over-reporters (ratio of energy intake to basal metabolic rate >2.40). However, the reason of using this approach is that it is considered to be the most practical method and individualized method of assessing plausibility of self-reported energy intake [33]. Moreover, it has been found that taking into consideration the reporting group and inclusion of a propensity score for misreporting was a useful tool to counteract attenuation of effect estimates [34]. According to this method, about 24.8% of the adolescents were considered as under-reporters and were included in the present study [33]. In addition, adolescents who were considered as over-reporters, 173 (4.9%), were excluded. According to the Goldberg et al. method [32], participants classified as underweight were excluded from analyses, as there were too few in this category to provide comparable information. Finally, 128 participants were also excluded due to missing data on confounders, such as moderate-to-vigorous physical activity level (MVPA).

### 2.3. Anthropometric Measurements

All anthropometric mensuration were collected using standard methodology [35]. Telescopic height-measuring instrument (model 225; SECA, Hamburg, Germany) was used to measure height to the nearest 0.1 cm, barefoot with the head oriented in the Frankfurt plan. Body weight was measured in underwear and without shoes by electronic scale (model 871; SECA, Hamburg, Germany) to the nearest 0.1 kg. The BMI was calculated by dividing body weight in kilograms by squared body height in meters. International Obesity Task Force criteria was used to classify the obesity status [36]. Children were classified into the normal weight, overweight, and obesity categories, based on pooled international data for BMI, and linked to the widely used adult obesity cut off point of 25 and 30 kg/m^2^ [36]. Additionally, skinfold thicknesses were measured in triplicate with a Holtain Caliper (Crymych, Wales, UK) from six body sites (triceps, subscapular, right side at biceps, suprailiac, thigh, and medial calf) to the nearest 0.2 mm, and the average of the three measures was used [37]. To obtain total body fat, the six skinfold thicknesses were summed. Body fat percentage was estimated from skinfold measurements, using the formula of Slaughter et al. [37]. The fat mass index (FMI) was calculated by dividing body fat mass in (kg) by the square of height in (m) [38]. Waist (WC) and hip (HC) circumference were also measured in triplicate to the nearest 0.1 cm with an anthropometric tape (SECA 200, Hamburg, Germany), and the average of the three measures was used. WC was measured at the midpoint between the lowest rib and the iliac crest [39] and considered a marker of abdominal fat. HC was measured at a level parallel to the floor, at the largest circumference of the buttocks.

### 2.4. Physical Activity Measurement

Accelerometers (Actigraph MTI, model GT1M, Manufacturing Technology Inc., Fort Walton Beach, FL, USA) were used to obtain an objective measurement of physical activity. The devices were placed on the lower back of the participants under the clothes using an elastic belt for seven sequent days. Instructions were given to participants when they wake up to wear the instrument and remove it for water-based activities and sleeping [40]. Data were downloaded to the computer using manufacturer software and analyzed later by software based on Visual Basic. Time spent in moderate and vigorous PA (MVPA) was determined using the cutoff point of 2000 cpm, to generate the various indices the number of days per week were multiplied by minutes per day to produce minutes per week for each activity [41]. More detailed information has been reported elsewhere [40].

### 2.5. Socioeconomic Status

A general questionnaire with socioeconomic status (SES), health outcomes, and nutritional status was fulfilled by the participants. Family affluence scale (FAS) was used as indicator of the adolescents’ material affluence; a recategorization into three levels included low, from 0 to 2; medium, from 3 to 5; and high, from 6 to 8 categories. In this study, FAS is considered a marker of SES. More detailed description about SES has been reported elsewhere [42].

### 2.6. Dietary Assessment

In order to determine the adolescent’s dietary intake, the HELENA Dietary Assessment Tool (HELENA–DIAT) was used. It is a self-administered computerized 24-h recall software, and it was developed and validated originally in Flemish adolescents and then in the HELENA–CSS [43]. HELENA–DIAT comprised the consumption from six meal occasions: breakfast, morning snack, lunch, afternoon snack, evening meal, and evening snack. It included two nonconsecutive 24 h recalls based on a weekday, with one week apart. A well-trained dietitian assessed the adolescents and helped them to complete the 24-h recall.

In total, about 800 photographs were available in the program. The participants could select visually from photographs the consumption amount and indicate the one more accurate with their actual intake. Furthermore, they could type in a textbox their intake amount from each food item. However, the participants can remove or modify the selected items at any time. In addition, for foods that could be sized by household measurements, like cups, several portions appeared on the screen, and the participants chose their consumption amount by clicking directly on the portion. In case the foods were eaten in combination with other food items, such as “French fries and mayonnaise” a box was shown on the screen to remind them to include this additional food item [44]. To calculate energy and nutrient intakes, data from HELENA–DIAT were linked to the German Food Code and Nutrient Database (Bundeslebensmittelschlüssel, version II.3.1) [45]. Taking into account occasionally consumed foods, the usual dietary intake of food and nutrients were estimated by multiple source method (MSM) [46]. The MSM first calculated the individual’s dietary intake and then built the population distribution based on the data. When the MSM method was applied, the dietary data were analyzed for average energy intake in kilocalories, kilojoules, and macronutrients (carbohydrates, proteins, and fat) in grams.

### 2.7. Food Grouping and Portion Size Calculation

Based on European food groups classification system, about 4179 foods and beverages, in the form of recipes or as individual food, were aggregated into 29 food groups [43,47]. As part of the general HELENA analysis, and based on the nutritional composition of food groups, some of them were further reaggregated for PS analysis, according to their nutritional value; for example, milk products and cheese were split to different food groups, due to the difference in their fat content. However, four food groups were excluded from the analysis: “products for special nutrition use,” “soya beverages,” “miscellaneous,” and “meat substitutes,” due to very low consumption (more than the 85% of the sample did not report consumption).

PS was established by the total intake of items in grams included in food group and consumed in the 24 h-recall divided by the number of eating occasions of these consumed items. In this study, the average amount was calculated from the two days included in the 24 h-recall by each meal occasion. For example, if an individual consumed 200 g of meat for lunch in the first day and 200 g in the lunch of the second day, then his/her PS at lunch from this food item was 200 g, and if individual consumed 200 g of meat only in lunch and did not consume meat in the lunch of second day, his/her PS was 200 g. Various studies have adapted the same methodology, such as the study of food PS effect on overweight in children and adults [48,49]. Thus, these data represent per-consumer averages, not per capita averages and are aimed to show the average change on the PS for those who consume a certain item. Thus, only participants who consumed a specific food group were included in the analysis.

### 2.8. Energy Dense Food Selection Criteria

PS were estimated for the 20% most frequently consumed food groups per eating occasion, and then we selected the food items that had been identified as high-ED foods, according to World Cancer Research Fund (containing 225–275 kcal/100 g) [20], and identified in previous research as the foods with the greatest contribution to energy intake, with positive associations to BMI in Europe and the rest of countries [11,48]. Eleven food groups were selected in this analysis and include 1—“breakfast cereals”; 2—“bread and rolls”; 3—“sweet bakery products”; 4—“confectionary nonchocolate”; 5—“chocolate”; 6—“sugar, honey, and jam”; 7—“cheese”; 8—“meat”; 9—“meat and poultry products”; 10—“vegetable oils”; and 11—“carbonated soft and isotonic drinks.”

### 2.9. Statistical Analysis

Normality was tested using the Kolmogorov–Smirnov method. Descriptive analyses of mean intakes (g) and standard deviation (SD) for general characteristics, energy, and macronutrients intake were presented. Student’s *t*- and chi-square (for categorial variables) tests were used to compare means of continuous variables by gender. Males and females develop different dietary patterns during the adolescent period, and males increase energy intake in order to increase satiety [50]. Another reason for splitting the analyses by gender is that changes in body composition are different, as males increase lean mass and females increase fat mass during their pubertal development. A stratified analysis was also carried out by splitting the sample into two groups, plausible reporters and under-reporters, to measure any potential differences of under-reporting on the associations under examination. In children, it was observed that consideration of the reporting group for misreporting turned out to be the most useful tool to counteract attenuation of effect estimates [34]. Student’s *t*-tests were performed to describe food PS for plausible reporters and under-reporters, and adolescents were stratified by weight status and between gender. To assess the relation between BMI, fat mass index (FMI), and food PS from each ED food group, multivariable linear regression analysis was carried out, using BMI and FMI as the dependent variables and food PS as the independent variable in both genders. Ordinal logistic regression models were carried out to determine the association between BMI categories (normal weight vs. overweight and obesity, combined) as dependent variable and PS from ED food groups between gender. Finally, a sensitivity analysis was carried out for all samples in order to detect any potential differences in the results after adjustment for misreporting. All regression analyses were adjusted for age, total energy intake (TEI), physical activity, and SES, because it was considered as important predictors of the outcome. Analyses were carried out using IBM–SPSS (v25, SPSS Inc., Chicago, IL, USA), and Stata (v13.0, College Station, TX: StataCorp LP, USA) was used for the multilevel logistic regression. *P*-values of 0.05 were used as representing statistical significance for all tests.

## 3. Results

### 3.1. General Characteristics of Study Participants

Sample descriptive characteristics by gender are presented in Table 1. A total of 1889 adolescents aged between 12.5 and 17.5 years old were included in this study. The plausible reporters number was *n* = 1421, and the under-reporters’ was *n* = 486. The majority (54.4%) of the participants were females. Generally, in all splitting groups, males had significantly higher waist circumference (*p* < 0.001), higher percentage of overweight (19.8%), and obesity (7.9%) (*p* < 0.001), and higher physical activity level (*p* < 0.001) than females. In contrast, females had significantly higher hip circumference (*p* = 0.002) and higher FMI, compared to males (*p* < 0.001). Furthermore, the results indicated that males had a significantly higher mean of TEI and higher mean intake from macronutrients (carbohydrate, protein, fat, and total sugar) than females (*p* < 0.001).

### 3.2. Portion Size Intake from Food Groups between BMI Categories in Both Genders

Table 2 shows the mean intake of food PS from ED food for plausible reporter and by BMI, normal weight or overweight/obesity in males and females. In males, the results indicate that plausible reporters with overweight/obesity had significantly higher portion mean intake from “cheese” and “carbonated soft drink,” compared with normal weight, while females with overweight/obesity had a higher portion mean intake from “bread and rolls,” and “confectionary nonchocolate,” compared with normal-weight females; In contrast, normal-weight females had a higher portion mean intake from “sweet bakery product,” compared with females with overweight or obesity. However, when misreporting was not considered in males, the results indicated that overweight/obesity participants had significantly higher portion mean intake from “cheese,” “meat and poultry products,” and “carbonated soft drink,” compared with normal weight. In females, when misreporting was not considered, normal weight had higher portion mean intake from “bread and rolls” and “sweet bakery products,” compared with overweight/obesity; meanwhile, females with overweight/obesity had significantly higher portion mean intake from “meat” and “confectionary nonchocolate,” compared with normal weight, when misreporting was not considered. Regarding under-reporters (Supplementary Table S1), males with overweight/obesity had significantly higher portion mean intake from “chocolate,” compared with normal weight males, while females with overweight/obesity had higher mean portion intake from “bread and rolls,” compared with normal weight.

### 3.3. Association between BMI and Portion Size of the Most Energy-Dense Foods

A positive association was observed between PS and BMI for some ED foods (Table 3). Consumption of higher PS from “breakfast cereals” was significantly associated (β = 0.012; 0.048) with BMI for males who were plausible reporters. When dietary misreporting was not considered, PS from “carbonated soft drink” was positively related with BMI (β = 0.002; *p* = 0.012), while PS from “sweet bakery products” were inversely associated with BMI (β = −0.04; *p* = 0.028). In females who were reported as plausible reporters dietary intake, PS from “sweet bakery product” were inversely associated with BMI (β = −0.004; *p* = 0.014). However, when dietary misreporting was not considered, PS from “sweet bakery product” and “chocolate” were inversely associated with BMI (β = −0.005; *p* = 0.002), (β = −0.007; *p* = 0.035), respectively. In under-reporters (Supplementary Table S2), higher PS of “bread and rolls” (β = 0.006; *p* ≤ 0.001), “chocolate” (β = 0.029; *p* = 0.028) and “sugar, honey, and jam” (β = 0.007; *p* = 0.012) were significantly associated with BMI in males. However, the results did not change when sensitivity analysis was carried out for all participants and with adjusting for misreporting.

### 3.4. Association between FMI and Portion Size of the Most Energy-Dense Foods

Table 4 shows the associations between FMI and PS of ED food groups by gender. In females who were plausible reporters and when misreporting was not considered, only PS from “sweet bakery product” (*p* < 0.050) were inversely related with FMI, while, in males and when misreporting was not considered, PS from “bread and rolls” (β = −0.006; *p* = 0.005) and “sweet bakery product” (β = −0.009; *p* = 0.002) showed an inversely significant relation to FMI. Regarding under-reporters (Supplementary Table S3), adolescent males showed a significant association between PS of “chocolate” and FMI (β = 0.061; *p* = 0.031). Meanwhile, in females, PS from “meat and poultry product” showed an inversely significant relation to FMI (β = −0.013; *p* = 0.023). When sensitivity analysis was carried out for all participants and with adjusting for misreporting, the results did not change.

### 3.5. Association between BMI Categories and Portion Size of the Most Energy-Dense Foods

Table 5 illustrates the results of ordinal logistic regression model by gender, using BMI categories (normal weight vs. overweight and obesity) as a dependent variable and PS of the most ED foods as independent variables. The model was adjusted for age, physical activity, TEI, and SES. Consumption of higher PS from “carbonated soft drinks” is associated with higher probability of having obesity in males who were plausible reporters (OR = 1.001; 95% CI 1.000; 1.002) and when misreporting was not considered (OR = 1.000; 95% CI 1.000; 1.001). Moreover, “sweet bakery products” is associated with lower probability of having obesity in males (OR = 0.996; 95% CI 0.993; 1.000) when misreporting was not considered. Consumption of higher PS from “bread and rolls” is associated with higher probability of having obesity in females who were plausible reporters (OR = 1.002; 95% CI 1.000; 1.004) and when misreporting was not considered (OR = 1.002; 95% CI 1.000; 1.003), while “sweet bakery products” is associated with lower probability of having obesity in females who were plausible reporters (OR = 0.996; 95% CI 0.991; 0.999) and when misreporting was not considered (OR = 0.996; 95% CI 0.992; 1.000). For under-reporting males (Supplementary Table S4), dietary intake of higher PS of “breakfast cereals” is associated with higher probability of having obesity (OR = 1.012; 95% CI 1.002; 1.024). However, the results did not change when sensitivity analysis was carried out for all participants and with adjusting for misreporting.

## 4. Discussion

The main results suggested that there is an association between PS of specific ED foods and BMI in adolescence. Specifically, in plausible reporters, “carbonated soft drinks” in males and “bread and rolls” in females are associated with a high probability of having obesity. Meanwhile, PS from “sweet bakery products” were associated with lower probability of having obesity in females, considering potential confounders, like physical activity level, TEI, and SES. This study also provides useful descriptive information on PS from ED food groups between gender and BMI categories in European adolescents.

Our results indicate that plausible reporters males with overweight or obesity had significantly higher portion mean intake from “cheese” and “carbonated soft drink,” compared with normal weight males. Plausible reporters’ females with obesity had higher mean portion intake from “bread and rolls” and “confectionary nonchocolate,” compared with normal weight females. Contrarily, a previous study based on adolescents showed that average PS of chocolate confectionery; cheese; and buns, cakes, and pastries were higher among normal weight than among adolescents with overweight or obesity [11]. Moreover, one study in adolescents noticed that energy intake from candy, packed goods, and ice cream was significantly greater in normal weight than in participants with obesity [51]. A study of adults from two national surveys found that mean PS of cakes, reported by French individuals with overweight/obesity, were 44% larger than normal-weight individuals; in contrast, adults with overweight/obesity reported smaller food PS from biscuit, crisps, and chocolate subgroups than normal-weight French adults [22]. However, food PS of biscuits reported by UK adults with overweight/obesity were 30% larger than those reported by normal-weight ones [22]. The possible reason of these results is that lean active subjects tend to select high energy and sugar diets, while subjects with overweight seem to prefer diets high in fat and restrict dietary sugars [52,53].

In this study, we adjusted for SES in all analyses. It is noteworthy that SES is considered as one of the strongest predictors of obesity development in all age groups and of living in a deprived area with oversized portions of unhealthy food [54]. In this context, a study in children reported larger food PS consumption of meat when the annual household income was higher [55]. Moreover, in adults, there has been found a small reliable relation between lower SES and consumption of large portions of unhealthy foods [54]. However, in our study, the observed associations were independent of SES, suggesting a strong association between PS from ED and BMI. It is also noteworthy that lower SES families are less likely to realize that their child is overweight and may believe that they should not impede the eating and activity behaviors of their child [56].

In this study, a positive association was observed between PS and BMI for some ED foods, and there were differences between plausible reporters and under-reporters. In another cross-sectional study in children, it was showed that overweight was positively correlated with the PS of sweetened pastries and biscuits [48]. Similarly, a positive correlation was found for PS of cakes, biscuits, and cheese and BMI in plausible reporter adolescents but not in the under-reporters. Meanwhile, the PS of high-fiber breakfast cereals was positively associated with BMI in under-reporters and among all adolescents [11]. The possible explanation of the positive association between breakfast cereals and BMI may be that some types of breakfast cereals contain nuts, sugars, honey, and fruit, which make the food more ED. In addition, we found that large PS from “bread and rolls,” “chocolate,” and “sugar, honey, and jam” groups were associated with higher BMI in under-reporter males. However, it was noticed that under-reporter children and adolescents were more likely to have overweight or obesity than normal reporters [57]. In addition, subjects with obesity tend to underreport their consumption to provide sociable desirable answers, even in adolescence [58]. It is noteworthy that several studies in children and adolescents found that one of the main reasons of dietary assessment errors was misreporting, mainly because of underreporting [59,60], which happens frequently in adolescents [61,62]. In this age, under-reporters generally provide lower intakes of ED foods and snacks than plausible reporters, because they tend to give socio-desirable answers [63], easily forget what they consumed, or/and they tend to have lower ability to report their own dietary intake [64]. In addition, adolescents tend to omit their food consumption by following a dietary restriction as a step to reduce their weight [64]. Moreover, it has been noticed that the exclusion of under-reporters or introducing them as covariates can strongly enhance the associations between dietary factors, including ED food and obesity [34].

In this study, we found that in females who were under-reporters on large PS from “meat and poultry product” showed an inverse significant relation with FMI. In a Korean study in children and adolescents, it was found that a high level of meat consumption was associated with lower BMI [65]. Contrarily, the possible explanation of an inverse relation between PS of “sweet bakery products” and BMI and FMI in plausible reporters females and “meat and poultry product” with BMI in under-reporter females is that adolescents with obesity tend to restrict their intake from sugars and fat foods as a primary step to reduce weight [66]. A hallmark of PS from these food groups is that they belong to the category of “convenience foods” or fast-food chains, which are sometimes packaged for single-serving consumption, and whose PS have been reported to be increasing, such as chocolate, bread, soda, and burgers [67]. In addition, researchers have noticed inverse or no associations with overweight and obesity, when the data were analyzed without adjustment for the ratio of (energy intake: estimated energy requirement), in both children and adolescents [68]. However, more studies are needed to give insights about our finding.

Regarding PS from “carbonated soft drinks,” we found in our study that large PS from this food group is associated with higher probability of having obesity in males. Clearly, in the last decades, PS of some foods, especially those consumed in restaurants, such as burgers and soda, have increased dramatically and concurrently with obesity prevalence [69]. Varied scientific reviews have confirmed the hypothesis that increased weight is linked with increased intake from carbonated soft drink in cross-sectional studies [70,71,72]. Moreover, it has been suggested from observational analysis that the odds of becoming obese over 5 years increased to 60% with each additional 12 ounces (340.19 g) of soda consumed per day by children [73], which means that the main contributor to the obesity epidemic is the elevating consumption of sweetened drinks [74]. The possible explanation of these results is that liquid carbohydrates, such as soda or solid jelly, cause less satiety, compared with solid carbohydrates sources; thus, increased consumption of energy-yielding fluids may enhance the positive energy balance [75,76]. However, in children, the trial studies that aim to reduce the effect of sugar-sweetened beverage intake on obesity are inconsistent; this may be due to failure to control confounders and some methodological limitations [77,78]. Although the effect sizes in our study are small, further studies are needed to confirm the association between food PS from ED food and obesity development.

In our study, a sensitivity analysis was carried out in order to detect any potential differences in the results after adjusting for misreporting, and the results did not change.

This study has some limitations. Firstly, although studies performed some years ago are not useful to describe the current situation, they are useful for assessing the associations between different types of variables. The information from holidays or from Fridays and Saturdays were not included, as the 24-h recalls were completed during school days. Secondly, the cross-sectional nature of HELENA study does not allow us to assess the behavior over a period of time and did not provide information in determining the cause-and-effect association. The self-reported questionnaires were used for collecting the food consumption data, and therefore, a social bias must be considered. However, a good agreement between the self-reports and the interviews was found [43]. Moreover, a high percentage of under- and over-reporters were detected. Additionally, the ED was not calculated from the food groups, so the main selected criteria for analysis were based on those foods identified in other studies as high sources of energy. However, there are several strengths in this study that need to be mentioned. To our best of knowledge, the present study is the first to investigate the association between the portion size of ED foods and body composition among European adolescents. Although residual confounding should be considered when interpreting the results, potential confounders such as age, total EI, physical activity, and SES were taken into account. Moreover, sample size had been selected from a wide geographical spread, including eight European cities, with large cultural dietary diversity. In order to increase the accuracy, highly standardized and validated procedures were used to collect the sample and assess anthropometric measurements.

## 5. Conclusions

In conclusion, large PS of “sweet bakery products” were found to be associated with a lower body composition among plausible females reporters, while increase PS from “breakfast cereals” groups were correlated with higher BMI in males. This was significant after adjustment for age, physical activity, TEI, and SES. Moreover, in subjects who were considered as plausible reporters, the results showed that large PS from “carbonated soft and isotonic drinks” in males and “bread and rolls” in females were associated with higher probability of having obesity, while large PS of “sweet bakery products” is associated with lower probability of having obesity in females. Further studies are needed to examine the prolonged exposure to large PS from several ED food sources and their effect on obesity development. If results are confirmed, this should be followed by nutritional health promotion programs directed to European adolescents to enhance their PS food selection.

## Figures and Tables

**Table 1:** Characteristics of all participants, plausible reporters, and under-reporters in study sample and mean daily intake of energy and macronutrient. Differences of mean values by gender were considered using Student’s *t*-test analysis.

	All Participants (*n* = 1889)			Plausible Reporters (*n* = 1421)			Under-Reporters (*n* = 468)		
General Characteristics	Males (*n* = 862)	Females (*n* = 1027)	*p*-Value	Males (*n* = 659)	Females (*n* = 761)	*p*-Value	Males (*n* = 202)	Females (*n* = 266)	*p*-Value
	M (SD)	M (SD)		M (SD)	M (SD)		M (SD)	M (SD)	
Age	14.8 (1.3)	14.7 (1.2)	0.181	14.7 (1.3)	14.7 (1.2)	0.408	14.8 (1.3)	14.8 (1.2)	0.199
Weight (kg)	63.6 (14.4)	57.2 (9.8)	**<0.001**	61.2 (12.9)	55.9 (9.1)	**<0.001**	71.2 (16.2)	60.8 (10.6)	**<0.001**
Height (cm)	170.2 (9.3)	162.3 (6.7)	**<0.001**	170.0 (9.3)	162.2 (6.9)	**<0.001**	170.8 (9.4)	162.8 (6.1)	**<0.001**
WC (cm)	75.1 (9.3)	71.1 (7.6)	**<0.001**	73.6 (8.4)	70.4 (7.0)	**0.001**	80.0 (10.4)	73.1 (8.8)	**0.004**
HC (cm)	91.3 (8.7)	93.7 (7.7)	**0.002**	89.9 (8.0)	92.8 (7.5)	**0.040**	95.9 (9.1)	96.2 (7.5)	**0.003**
BMI (kg/m^2^)	21.8 (3.8)	21.7 (3.3)	**0.001**	21.0 (3.3)	21.2 (3.1)	0.068	24.2 (4.3)	22.9 (3.5)	**0.014**
BMI Categories (*n*, %) Normal weight Overweight Obese	623 (72.3%) 171 (19.8%) 68 (7.9%)	814 (79.3%) 172 (16.7%) 41 (4.0%)	**<0.001**	530 (80.4%) 97 (14.7%) 32 (4.9%)	642 (84.4%) 96 (12.6%) 23 (3.0%)	**<0.001**	92 (45.6%) 74 (36.6%) 36 (17.8%)	172 (64.7%) 76 (28.5%) 18 (6.8%)	**<0.001**
FMI (kg/m^2^)	7.8 (6.4)	9.6 (4.2)	**<0.001**	6.9 (5.5)	9.1 (3.9)	**<0.001**	10.8 (8.1)	10.9 (4.5)	**<0.001**
MVPA (min/week)	804.50 (607.26)	642.75 (523.07)	**<0.001**	801.42 (599.30)	618.24 (518.46)	**<0.001**	816.97 (634.58)	712.90 (530.89)	**0.006**
SES categories (*n*, %) Low Medium High	74 (8.6%) 487 (56.8%) 296 (34.6%)	119 (11.7%) 558 (54.5%) 346 (33.8%)	0.207	48 (7.3%) 382 (58.2%) 226 (34.5%)	92 (12.1%) 419 (55.2%) 248 (32.7%)	0.051	26 (13.0%) 105 (54.5%) 69 (34.5%)	27 (10.2%) 139 (52.6%) 98 (37.2%)	0.221
Total energy intake (kcal)	2348.00 (653.05)	1848.01 (487.20)	**<0.001**	2691.33 (440.93)	2128.10 (301.58)	**<0.001**	1578.39 (312.04)	1267.16 (221.76)	**<0.001**
Carbohydrates (g)	285.58 (89.65)	228.13 (68.43)	**<0.001**	323.85 (71.23)	260.14 (52.36)	**<0.001**	195.25 (49.64)	158.86 (36.33)	**<0.001**
% of energy from carbohydrates	48.79 (7.62)	49.49 (7.47)	0.657	48.06 (6.35)	48.80 (6.15)	0.633	49.45 (7.85)	50.20 (7.68)	0.865
Proteins (g)	92.86 (28.20)	70.99 (20.13)	**<0.001**	103.13 (23.43)	79.78 (15.97)	**<0.001**	69.32 (18.48)	52.92 (13.07)	**<0.001**
% of energy from proteins	16.11 (3.50)	15.61 (3.12)	**0.004**	15.38 (2.79)	15.02 (2.43)	**0.004**	17.69 (3.72)	16.81 (3.41)	**0.616**
Fat (g)	91.79 (32.02)	73.04 (24.14)	**<0.001**	106.11 (24.92)	84.94 (18.16)	**<0.001**	59.23 (17.35)	48.47 (12.29)	**<0.001**
% of energy from fat	34.99 (6.46)	34.99 (6.46)	0.455	35.35 (5.15)	35.84 (5.03)	0.585	33.80 (7.27)	34.41 (6.22)	0.348
Total sugars (g)	136.52 (57.69)	111.24 (45.12)	**<0.001**	152.25 (55.33)	124.99 (43.54)	**<0.001**	93.95 (37.36)	79.59 (28.51)	**<0.001**
% of energy from total sugars	23.42 (7.64)	24.32 (7.87)	0.611	22.62 (7.06)	23.48 (7.28)	0.369	23.76 (7.97)	25.26 (8.04)	0.453

M: mean, SD: standard deviation, BMI: body mass index, WC: waist circumference, HC: hip circumference, MVPA: moderate-to-vigorous physical activity level, FMI: fat mass index, socioeconomic status (SES): socioeconomic status, Boldface values indicate significance, *p*-value < 0.05.

**Table 2:** Mean portion size of energy-dense (ED) foods by BMI status in both genders, for consumers of different food categories, using *t*-test.

	Plausible Reporters (*n* = 1421)										All Participants (*n* = 1889)									
Body Mass Index Categories	Males				*p*-Value	Females				*p*-Value	Males				*p*-Value	Females				*p*-Value
	Normal Weight (*n* = 530)		Overweight/Obesity (*n* = 129)			Normal Weight (*n* = 642)		Overweigh/Obesity (*n* = 119)			Normal Weight (*n* = 623)		Overweight/Obesity (*n* = 236)			Normal Weight (*n* = 814)		Overweight/Obesity (*n* = 213)		
Food Groups (g)	n	M (SD)	n	M (SD)		n	M (SD)	n	M (SD)		n	M (SD)	n	M (SD)		n	M (SD)	n	M (SD)	
Breakfast cereals	151	53.99 (36.23)	24	54.42 (29.93)	0.680	175	42.81 (24.92)	20	38.30 (30.28)	0.275	167	51.93 (35.36)	40	61.63 (85.94)	0.066	198	42.11 (24.37)	41	37.71 (26.28)	0.442
Bread and rolls	487	170.18 (111.77)	114	176.80 (103.42)	0.709	611	142.45 (100.58)	111	162.07 (119.61)	**0.003**	562	162.48 (109.22)	209	150.69 (99.91)	0.435	756	132.59 (95.47)	195	131.99 (107.56)	**0.003**
Sweet bakery product	265	129.66 (101.86)	50	107.017 (80.82)	0.075	384	108.18 (91.15)	68	79.64 (58.45)	**0.022**	298	123.75 (98.42)	79	90.82 (72.35)	**0.006**	444	103.58 (87.94)	97	75.38 (57.15)	**0.005**
Confectionary nonchocolate	77	34.45 (42.66)	12	40.12 (54.70)	0.879	181	24.76 (27.29)	29	34.52 (55.37)	**0.001**	90	33.36 (41.19)	20	39.42 (51.27)	0.240	209	24.94 (26.68)	49	31.24 (45.99)	**0.033**
Chocolate	161	63.46 (75.91)	33	58.899 (71.18)	0.745	216	47.40 (51.21)	29	41.65 (48.99)	0.387	178	60.91 (73.02)	45	61.58 (67.68)	0.922	251	45.91 (49.62)	49	35.02 (41.58)	0.132
Sugar, honey, and jam	98	30.03 (23.67)	25	28.06 (19.70)	0.911	135	23.77 (24.22)	30	15.46 (12.80)	0.226	110	29.10 (22.98)	36	34.10 (32.07)	0.260	156	23.38 (23.32)	41	16.36 (13.39)	0.251
Cheese	208	49.10 (61.41)	41	53.22 (38.63)	**0.007**	217	36.06 (29.94)	38	35.73 (28.25)	0.990	230	48.27 (59.54)	72	75.72 (57.11)	**0.024**	265	34.14 (28.47)	60	31.80 (25.18)	0.761
Meat	289	168.05 (172.14)	46	185.94 (229.92)	0.280	289	120.30 (126.28)	44	142.53 (150.17)	0.178	323	169.07 (173.77)	102	169.07 (173.77)	0.850	363	112.46 (121.32)	83	130.22 (169.36)	**0.049**
Meat and poultry product	395	125.88 (123.40)	93	149.26 (125.03)	0.158	452	92.43 (85.89)	83	83.96 (71.46)	0.461	445	122.29 (121.60)	167	185.44 (153.84)	**0.017**	545	88.36 (83.06)	134	78.71 (71.81)	0.236
Vegetable oils	111	21.96 (18.04)	43	20.56 (18.14)	0.891	149	17.10 (12.86)	34	19.21 (17.71)	0.159	123	21.48 (17.78)	58	19.63 (16.97)	0.858	188	16.55 (13.22)	65	18.57 (15.13)	0.316
Carbonated soft and isotonic drink	229	442.40 (271.99)	49	525.41 (389.77)	**0.009**	250	392.24 (310.88)	27	400.51 (244.72)	0.570	253	452.19 (284.26)	82	557.73 (439.49)	**0.001**	284	384.25 (299.28)	50	419.51 (282.55)	0.912

*n*: number of consumers, M: mean, SD: standard deviation, Boldface values indicate significance, *p*-value < 0.05.

**Table 3:** The association between BMI and portion sizes (PS) of most ED food using multiple linear regression model.

Body Mass Index *	Plausible Reporters (*n* = 1421)								All Participants (*n* = 1889)							
	Males				Females				Males				Females			
Food Groups (g)	β	95% CI		*p*-Value	β	95% CI		*p*-Value	β	95% CI		*p*-Value	β	95% CI		*p*-Value
		Upper	Lower			Upper	Lower			Upper	Lower			Upper	Lower	
Breakfast cereals	0.012	0.000	0.023	**0.048**	−0.001	−0.016	0.014	0.892	0.006	−0.002	0.017	0.154	−0.003	−0.019	0.013	0.698
Bread and rolls	0.001	−0.001	0.004	0.223	0.002	0.000	0.004	0.069	0.000	−0.002	0.003	0.880	−0.001	−0.003	0.001	0.978
Sweet bakery product	−0.001	−0.004	0.002	0.548	−0.004	−0.008	−0.001	**0.014**	−0.004	−0.008	0.000	**0.028**	−0.005	−0.008	−0.002	**0.002**
Confectionary nonchocolate	0.004	−0.001	0.018	0.613	0.006	−0.006	0.017	0.318	0.001	−0.014	0.015	0.923	0.003	−0.009	0.015	0.597
Chocolate	−0.001	−0.007	0.005	0.819	−0.003	−0.009	0.003	0.291	0.003	−0.006	0.006	0.990	−0.007	−0.014	0.000	**0.035**
Sugar, honey, and jam	−0.001	−0.031	0.028	0.922	−0.013	−0.034	0.008	0.218	0.026	0.000	0.052	0.051	−0.014	−0.034	0.007	0.186
Cheese	0.004	−0.003	0.011	0.246	0.001	−0.011	0.013	0.905	0.001	−0.002	0.005	0.357	−0.004	−0.016	0.008	0.550
Meat	0.001	−0.001	0.003	0.300	0.001	−0.002	0.003	0.663	0.000	−0.002	0.002	0.874	0.000	−0.005	0.002	0.366
Meat and poultry product	0.001	−0.001	0.004	0.213	−0.001	−0.004	0.002	0.493	0.000	0.000	0.001	0.390	−0.001	−0.004	0.001	0.327
Vegetable oils	0.004	−0.031	0.039	0.812	−0.007	−0.044	0.030	0.692	−0.004	−0.038	0.030	0.825	−0.002	−0.034	0.030	0.914
Carbonated soft and isotonic drink	0.001	0.000	0.002	0.067	0.001	0.000	0.002	0.241	0.002	0.000	0.003	**0.012**	0.001	0.000	0.002	0.122

β: regression coefficient. CI: confidence interval, BMI: body mass index. * Adjusting for confounders: age, Moderate-to-vigorous physical activity (MVPA), and total energy intake (TEI) and SES. Boldface values indicate significance, *p*-value < 0.05.

**Table 4:** The association between FMI and portion size of most ED food between gender using multiple linear regression model.

Fat Mass Index *	Plausible Reporters (*n* = 1421)								All Participants (*n* = 1889)							
	Males				Females				Males				Females			
Food Groups (g)	β	95% CI		*p*-Value	β	95% CI		*p*-Value	β	95% CI		*p*-Value	β	95% CI		*p*-Value
		Upper	Lower			Upper	Lower			Upper	Lower			Upper	Lower	
Breakfast cereals	0.011	−0.080	0.029	0.246	−0.008	−0.026	0.009	0.345	0.006	−0.008	0.019	0.417	−0.014	−0.033	0.005	0.156
Bread and rolls	−0.003	−0.007	0.001	0.165	0.000	−0.002	0.003	0.817	−0.006	−0.010	−0.002	**0.005**	−0.002	−0.005	0.001	0.122
Sweet bakery product	−0.005	−0.011	0.000	0.064	−0.006	−0.010	−0.002	**0.005**	−0.009	−0.015	−0.003	**0.002**	−0.007	−0.011	−0.003	**0.001**
Confectionary nonchocolate	0.013	−0.010	0.036	0.281	0.009	−0.005	0.024	0.194	0.014	−0.010	0.038	0.240	0.008	−0.006	0.023	0.257
Chocolate	−0.004	−0.014	0.006	0.408	−0.002	−0.010	0.005	0.554	−0.002	−0.012	0.008	0.756	−0.004	−0.013	0.004	0.328
Sugar, honey, and jam	−0.007	−0.046	0.032	0.730	−0.020	−0.045	0.006	0.124	−0.013	−0.048	0.022	0.471	−0.018	−0.044	0.008	0.173
Cheese	0.001	−0.011	0.013	0.883	0.004	−0.012	0.021	0.605	0.003	−0.002	0.008	0.305	−0.001	−0.017	0.015	0.900
Meat	0.002	−0.001	0.005	0.144	0.002	−0.001	0.005	0.176	0.001	−0.002	0.004	0.518	0.002	−0.001	0.005	0.225
Meat and poultry product	−0.001	−0.005	0.003	0.695	0.000	−0.004	0.005	0.820	0.000	−0.001	0.001	0.587	−0.003	−0.007	0.001	0.196
Vegetable oils	0.007	−0.059	0.073	0.836	0.006	−0.041	0.053	0.815	−0.006	−0.069	0.057	0.847	0.019	−0.023	0.062	0.372
Carbonated soft and isotonic drink	0.001	−0.001	0.003	0.167	0.001	−0.001	0.002	0.296	0.001	−0.001	0.002	0.585	0.001	−0.001	0.002	0.427

β: regression coefficient. CI: confidence interval, FMI: fat mass index. * Adjusting for confounders: age, physical activity (MVPA), TEI, and SES. Boldface values indicate significance, *p*-value < 0.05.

**Table 5:** Ordinal logistic regression model, the association between BMI categories and ED food portion groups between gender.

BMI Categories *	Plausible Reporters (*n* = 1421)								All Participants (*n* = 1889)							
	Males				Females				Males				Females			
Food Groups (g)	OR	95% CI		*p*-Value	OR	95% CI		*p*-Value	OR	95% CI		*p*-Value	OR	95% CI		*p*-Value
		Upper	Lower			Upper	Lower			Upper	Lower			Upper	Lower	
Breakfast cereals	1.002	0.989	1.015	0.751	0.999	0.978	1.020	0.912	1.004	0.998	1.010	0.152	0.998	0.981	1.015	0.802
Bread and rolls	1.000	0.999	1.002	0.608	1.002	1.000	1.004	**0.012**	1.001	0.999	1.002	0.358	1.002	1.000	1.003	**0.047**
Sweet bakery product	0.997	0.994	1.000	0.150	0.996	0.991	0.999	**0.046**	0.996	0.993	1.000	**0.049**	0.996	0.992	1.000	**0.032**
Confectionary nonchocolate	1.004	0.992	1.017	0.506	1.008	0.999	1.018	0.123	1.006	0.995	1.016	0.305	1.006	0.997	1.015	0.174
Chocolate	0.999	0.993	1.004	0.666	0.999	0.990	1.008	0.854	1.000	0.996	1.006	0.782	0.997	0.989	1.006	0.488
Sugar, honey, and jam	0.993	0.972	1.014	0.514	0.975	0.941	1.010	0.161	1.009	0.995	1.023	0.233	0.981	0.955	1.008	0.165
Cheese	1.001	0.996	1.006	0.665	1.003	0.991	1.015	0.659	1.000	0.999	1.002	0.238	1.001	0.990	1.012	0.886
Meat	1.000	0.999	1.002	0.613	1.001	0.999	1.002	0.525	1.000	0.999	1.002	0.535	1.001	0.999	1.003	0.284
Meat and poultry product	1.001	0.999	1.003	0.102	0.999	0.996	1.002	0.531	1.000	0.999	1.000	0.292	0.999	0.996	1.002	0.440
Vegetable oils	0.995	0.974	1.016	0.651	1.006	0.982	1.031	0.636	0.995	0.975	1.015	0.603	1.012	0.993	1.032	0.221
Carbonated soft and isotonic drink	1.001	1.000	1.002	**0.032**	1.000	0.999	1.002	0.636	1.000	1.000	1.001	**0.035**	1.000	0.999	1.002	0.262

OR: odd ratio. CI: confident interval. * Adjusting for confounders: age, moderate-to-vigorous physical activity (MVPA), and TEI and SES. Boldface values indicate significance, *p*-value < 0.05.

## Data Availability

The data presented in this study are available for further scientific analysis on request from the coordinator of the HELENA study to the following e-mail: lmoreno@unizar.es.

## Supplementary Materials

The following are available online at https://www.mdpi.com/2072-6643/13/3/954/s1, Table S1: Mean intake of food PS from ED food for plausible reporter and by BMI, normal weight or overweight/obesity in males and females. Table S2: The association between BMI and PS of most ED food in under-reporters, using multiple linear regression model. Table S3: The association between FMI and portion size of most ED food between gender in under-reporters, using multiple linear regression model. Table S4: Ordinal logistic regression model, the association between BMI categories and ED food portion groups in under-reporters and between gender.

## HELENA Study Group

Coordinator: Luis A. Moreno.

Core Group members: Luis A. Moreno, Fréderic Gottrand, Stefaan De Henauw, Marcela González-Gross, Chantal Gilbert.

Steering Committee: Anthony Kafatos (President), Luis A. Moreno, Christian Libersa, Stefaan De Henauw, Sara Castelló, Fréderic Gottrand, Mathilde Kersting, Michael Sjöstrom, Dénes Molnár, Marcela González-Gross, Jean Dallongeville, Chantal Gilbert, Gunnar Hall, Lea Maes, Luca Scalfi.

Project Manager: Pilar Meléndez.

1. Universidad de Zaragoza (Spain):
  Luis A. Moreno, José A. Casajús, Jesús Fleta, Gerardo Rodríguez, Concepción Tomás, María I. Mesana, Germán Vicente-Rodríguez, Adoración Villarroya, Carlos M. Gil, Ignacio Ara, Juan Fernández Alvira, Gloria Bueno, Olga Bueno, Juan F. León, Jesús Mª Garagorri, Idoia Labayen, Iris Iglesia, Silvia Bel, Luis A. Gracia Marco, Theodora Mouratidou, Alba Santaliestra-Pasías, Iris Iglesia, Esther González-Gil, Pilar De Miguel-Etayo, Mary Miguel-Berges, Isabel Iguacel, Azahara Rupérez.
2. Consejo Superior de Investigaciones Científicas (Spain):
  Ascensión Marcos, Julia Wärnberg, Esther Nova, Sonia Gómez, Ligia Esperanza Díaz, Javier Romeo, Ana Veses, Belén Zapatera, Tamara Pozo, David Martínez.
3. Université de Lille 2 (France):
  Laurent Beghin, Christian Libersa, Frédéric Gottrand, Catalina Iliescu, Juliana Von Berlepsch.
4. Research Institute of Child Nutrition Dortmund, Rheinische Friedrich–Wilhelms–Universität Bonn (Germany):
  Mathilde Kersting, Wolfgang Sichert-Hellert, Ellen Koeppen.
5. Pécsi Tudományegyetem (University of Pécs) (Hungary):
  Dénes Molnar, Eva Erhardt, Katalin Csernus, Katalin Török, Szilvia Bokor, Mrs. Angster, Enikö Nagy, Orsolya Kovács, Judit Répasi.
6. University of Crete School of Medicine (Greece):
  Anthony Kafatos, Caroline Codrington, María Plada, Angeliki Papadaki, Katerina Sarri, Anna Viskadourou, Christos Hatzis, Michael Kiriakakis, George Tsibinos, Constantine Vardavas, Manolis Sbokos, Eva Protoyeraki, Maria Fasoulaki.
7. Institut für Ernährungs- und Lebensmittelwissenschaften–Ernährungphysiologie. Rheinische Friedrich Wilhelms Universität (Germany):
  Peter Stehle, Klaus Pietrzik, Marcela González-Gross, Christina Breidenassel, Andre Spinneker, Jasmin Al-Tahan, Miriam Segoviano, Anke Berchtold, Christine Bierschbach, Erika Blatzheim, Adelheid Schuch, Petra Pickert.
8. University of Granada (Spain):
  Manuel J. Castillo, Ángel Gutiérrez, Francisco B Ortega, Jonatan R Ruiz, Enrique G Artero, Vanesa España, David Jiménez-Pavón, Palma Chillón, Cristóbal Sánchez-Muñoz, Magdalena Cuenca.
9. Istituto Nazionalen di Ricerca per gli Alimenti e la Nutrizione (Italy):
  Davide Arcella, Elena Azzini, Emma Barrison, Noemi Bevilacqua, Pasquale Buonocore, Giovina Catasta, Laura Censi, Donatella Ciarapica, Paola D’Acapito, Marika Ferrari, Myriam Galfo, Cinzia Le Donne, Catherine Leclercq, Giuseppe Maiani, Beatrice Mauro, Lorenza Mistura, Antonella Pasquali, Raffaela Piccinelli, Angela Polito, Romana Roccaldo, Raffaella Spada, Stefania Sette, Maria Zaccaria.
10. University of Napoli “Federico II” Dept of Food Science (Italy):
  Luca Scalfi, Paola Vitaglione, Concetta Montagnese.
11. Ghent University (Belgium):
  Ilse De Bourdeaudhuij, Stefaan De Henauw, Tineke De Vriendt, Lea Maes, Christophe Matthys, Carine Vereecken, Mieke de Maeyer, Charlene Ottevaere, Inge Huybrechts.
12. Medical University of Vienna (Austria):
  Kurt Widhalm, Katharina Phillipp, Sabine Dietrich, Birgit Kubelka, Marion Boriss-Riedl.
13. Harokopio University (Greece):
  Yannis Manios, Eva Grammatikaki, Zoi Bouloubasi, Tina Louisa Cook, Sofia Eleutheriou, Orsalia Consta, George Moschonis, Ioanna Katsaroli, George Kraniou, Stalo Papoutsou, Despoina Keke, Ioanna Petraki, Elena Bellou, Sofia Tanagra, Kostalenia Kallianoti, Dionysia Argyropoulou, Stamatoula Tsikrika, Christos Karaiskos.
14. Institut Pasteur de Lille (France):
  Jean Dallongeville, Aline Meirhaeghe.
15. Karolinska Institutet (Sweden):
  Michael Sjöstrom, Jonatan R Ruiz, Francisco B. Ortega, María Hagströmer, Anita Hurtig Wennlöf, Lena Hallström, Emma Patterson, Lydia Kwak, Julia Wärnberg, Nico Rizzo.
16. Asociación de Investigación de la Industria Agroalimentaria (Spain):
  Jackie Sánchez-Molero, Sara Castelló, Elena Picó, Maite Navarro, Blanca Viadel, José Enrique Carreres, Gema Merino, Rosa Sanjuán, María Lorente, María José Sánchez.
17. Campden BRI (United Kingdom):
  Chantal Gilbert, Sarah Thomas, Elaine Allchurch, Peter Burgess.
18. SIK—Institutet foer Livsmedel och Bioteknik (Sweden):
  Gunnar Hall, Annika Astrom, Anna Sverkén, Agneta Broberg.
19. Meurice Recherche & Development asbl (Belgium):
  Annick Masson, Claire Lehoux, Pascal Brabant, Philippe Pate, Laurence Fontaine.
20. Campden & Chorleywood Food Development Institute (Hungary):
  Andras Sebok, Tunde Kuti, Adrienn Hegyi.
21. Productos Aditivos SA (Spain):
  Cristina Maldonado, Ana Llorente.
22. Cárnicas Serrano SL (Spain):
  Emilio García.
23. Cederroth International AB (Sweden):
  Holger von Fircks, Marianne Lilja Hallberg, Maria Messerer.
24. Lantmännen Food R&D (Sweden):
  Mats Larsson, Helena Fredriksson, Viola Adamsson, Ingmar Börjesson.
25. European Food Information Council (Belgium):
  Laura Fernández, Laura Smillie, Josephine Wills.
26. Universidad Politécnica de Madrid (Spain):
  Marcela González-Gross, Raquel Pedrero-Chamizo, Agustín Meléndez, Jara Valtueña, David Jiménez-Pavón, Ulrike Albers, Pedro J. Benito, Juan José Gómez Lorente, David Cañada, Alejandro Urzanqui, Rosa María Torres, Paloma Navarro.
