# Supplementary material for: The Association between Portion Sizes from High-Energy-Dense Foods and Body Composition in European Adolescents: The HELENA Study
Source: Nutrients. 2021 Mar 16;13(3):954. doi: 10.3390/nu13030954 (PMC7998698; doi:10.3390/nu13030954)
Supplement: Supplementary file 1 [file nutrients-13-00954-s001.pdf]

Table S1: Mean intake of food PS from ED food for under-reporters and by BMI, normal weight or overweight/obesity in males and females.

	**Under-reporters**									
**Body Mass Index categories ***	Males				*p-value*	Females				*p-value*
	**Normal weight (*n*=92)**		**Overweight/ Obesity (*n*=110)**			**Normal weight (*n*=172)**		**Overweight/ Obesity (*n*=94)**		
**Food groups (g)**	*n*	M (SD)	*n*	M (SD)		*n*	M (SD)	*n*	M (SD)	
**Breakfast cereals**	16	32.47 (16.19)	16	72.44 (132.74)	0.068	23	36.80 (19.28)	21	37.14 (22.56)	0.520
**Bread and rolls**	75	112.50 (73.94)	95	119.35 (86.09)	0.070	145	91.02 (52.61)	84	92.25 (72.62)	**0.001**
**Sweet bakery product**	33	76.35 (41.20)	29	62.90 (43.29)	0.400	60	74.16 (55.76)	29	65.40 (53.63)	0.296
**Confectionary non chocolate**	13	26.87 (31.74)	8	38.38 (49.29)	0.206	28	26.07 (22.79)	20	26.49 (28.03)	0.845
**Chocolate**	17	36.82 (26.31)	12	68.96 (59.18)	**0.018**	35	36.70 (37.65)	20	25.41 (25.79)	0.418
**Sugar, honey, and Jam**	12	21.50 (14.99)	11	47.82 (48.69)	0.160	21	20.87 (16.59)	11	18.83 (17.09)	0.943
**Cheese**	22	40.39 (37.63)	31	105.48 (390.96)	0.166	48	25.44 (18.39)	22	24.99 (17.28)	0.898
**Meat**	34	177.74 (189.62)	56	155.63 (162.08)	0.656	74	81.83 (94.22)	39	116.34 (189.75)	0.072
**Meat and poultry product**	49	94.83 (81.89)	74	230.91 (278.42)	0.182	93	68.57 (64.45)	51	70.15 (72.25)	0.718
**Vegetable oils**	12	17.06 (15.11)	15	16.95 (13.26)	0.729	39	14.45 (14.48)	31	17.87 (11.95)	0.989
**Carbonated soft and isotonic drink**	24	545.57 (376.74)	33	605.72 (507.02)	0.215	34	325.51 (186.62)	23	441.82 (325.69)	0.066

n: number of consumers, M: mean, SD: standard deviation, Boldface values indicate significance, *p*-value <0.05.

Table S2: The association between BMI and PS of most ED food in under-reporters, using multiple linear regression model.

**Body Mass Index***	**Under-reporters**							
	**Males**				**Females**			
**Food groups (g)**	β	95% CI		*p-value*	β	95% CI		*p-value*
		Upper	Lower			Upper	Lower	
**Breakfast cereals**	0.002	-0.011	0.015	0.751	0.011	-0.045	0.066	0.705
**Bread and rolls**	0.014	0.006	0.022	**<0.001**	0.001	-0.005	0.005	0.805
**Sweet bakery product**	-0.016	-0.039	0.008	0.181	-0.008	-0.020	0.005	0.238
**Confectionary non chocolate**	-0.011	-0.057	0.035	0.614	-0.018	-0.057	0.022	0.366
**Chocolate**	0.029	0.003	0.054	**0.028**	-0.023	-0.052	0.006	0.119
**Sugar, honey, and Jam**	0.007	0.018	0.135	**0.012**	-0.010	-0.085	0.064	0.778
**Cheese**	0.003	-0.010	0.028	0.525	-0.005	-0.049	0.038	0.815
**Meat**	-0.002	-0.008	0.003	0.465	0.001	-0.004	0.005	0.774
**Meat and poultry product**	0.001	-0.001	0.001	0.847	0.004	-0.005	0.012	0.417
**Vegetable oils**	-0.031	-0.152	0.090	0.604	0.032	-0.040	0.104	0.380
**Carbonated soft and isotonic drink**	0.001	-0.003	0.004	0.735	0.003	-0.001	0.006	0.108

β: regression coefficient. CI: confidence interval, BMI: body mass index. *Adjusting for confounders: age, Moderate-to-vigorous physical activity (MVPA), and TEI and SES. Boldface values indicate significance, *p*-value <0.05.

Table S3: The association between FMI and portion size of most ED food between gender in under-reporters using multiple linear regression model.

**Fat Mass Index***	**Under-reporters**							
	**Males**				**Females**			
**Food groups (g)**	β	95% CI		*p-value*	β	95% CI		*p-value*
		Upper	Lower			Upper	Lower	
**Breakfast cereals**	0.002	-0.022	0.026	0.874	-0.014	-0.080	0.052	0.668
**Bread and rolls**	0.001	-0.013	0.014	0.935	-0.005	-0.015	0.004	0.272
**Sweet bakery product**	-0.024	-0.068	0.020	0.282	-0.012	-0.029	0.005	0.173
**Confectionary non chocolate**	0.028	-0.062	0.118	0.519	0.000	-0.049	0.049	0.991
**Chocolate**	0.061	0.006	0.117	**0.031**	-0.006	-0.048	0.036	0.776
**Sugar, honey, and Jam**	-0.030	-0.125	0.064	0.510	0.017	-0.088	0.122	0.746
**Cheese**	0.002	-0.005	0.008	0.584	0.004	-0.052	0.059	0.890
**Meat**	-0.002	-0.012	0.007	0.633	0.003	-0.003	0.010	0.280
**Meat and poultry product**	0.000	-0.001	0.002	0.769	-0.013	-0.025	-0.002	**0.023**
**Vegetable oils**	-0.036	-0.250	0.178	0.731	0.066	-0.029	0.162	0.172
**Carbonated soft and isotonic drink**	-0.003	-0.008	0.002	0.197	0.004	-0.005	0.003	0.984

β: regression coefficient. CI: confidence interval, FMI: fat mass index. *Adjusting for confounders: age, physical activity (MVPA), TEI, and SES. Boldface values indicate significance, *p*-value <0.05.

Table S4: Ordinal logistic regression model, the association between BMI categories and ED food portion groups in under-reporters and between gender.

**Body Mass Index categories***	**Under-reporters**							
	**Males**				**Females**			
**Food groups (g)**	OR	95% CI		*p-value*	OR	95% CI		*p-value*
		Upper	Lower			Upper	Lower	
**Breakfast cereals**	1.012	1.002	1.024	**0.023**	0.987	0.957	1.018	0.411
**Bread and rolls**	1.002	0.998	1.006	0.260	0.999	0.995	1.004	0.702
**Sweet bakery product**	0.991	0.979	1.003	0.147	0.996	0.987	1.006	0.460
**Confectionary non chocolate**	0.997	0.970	1.025	0.822	1.000	0.976	1.025	0.963
**Chocolate**	1.012	0.991	1.033	0.256	0.985	0.961	1.010	0.251
**Sugar, honey, and Jam**	1.037	0.982	1.096	0.189	0.988	0.942	1.036	0.611
**Cheese**	1.000	0.999	1.002	0.683	0.985	0.953	1.018	0.366
**Meat**	0.999	0.996	1.001	0.282	1.001	0.999	1.004	0.346
**Meat and poultry product**	1.000	0.999	1.000	0.822	0.998	0.993	1.004	0.545
**Vegetable oils**	0.996	0.941	1.055	0.902	1.032	0.993	1.073	0.113
**Carbonated soft and isotonic drink**	1.000	0.999	1.001	0.687	1.000	0.999	1.003	0.483

OR: odd ratio. CI: confident interval. *Adjusting for confounders: age, moderate-to-vigorous physical activity (MVPA), and TEI and SES. Boldface values indicate significance, *p*-value <0.05.
